# Molecular insights into desmoid tumors

**DOI:** 10.18632/oncotarget.21293

**Published:** 2017-09-28

**Authors:** Nam Bui, Shivaani Kummar

**Affiliations:** Shivaani Kummar: Division of Medical Oncology, Stanford Univeristy School of Medicine, Palo Alto, CA, USA

**Keywords:** NICD, molecular insights

Desmoid tumors are rare, soft tissue tumors with no known capacity for metastasis. Although considered a benign lesion, desmoids can be locally aggressive and cause significant morbidity due to mass effect on adjacent structures and organs. Sites of involvement generally are the extremities, trunk, abdominal wall, and the intra-abdominal region. Risk factors include induced situations such as high estrogen states (pregnancy) and antecedent trauma, or germline predispositions such as the familial adenomatous polyposis (FAP) syndrome.

**Figure 1:**
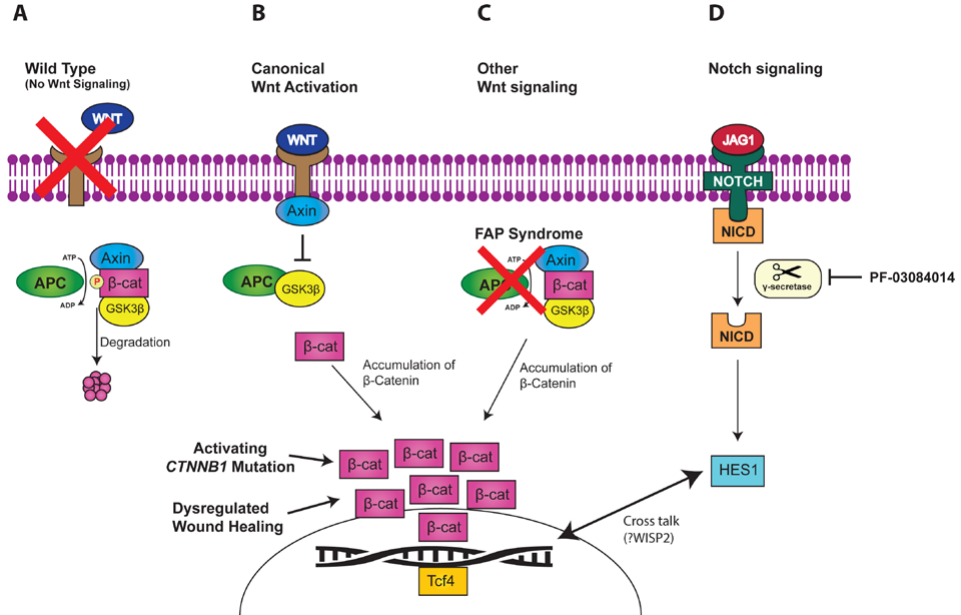
Wnt and Notch signaling pathways **A.** *APC* mediated β-catenin degradation. **B.** Canonical Wnt activation leading to β-catenin accumulation. **C.** *APC* inactivation leading to β-catenin accumulation **D.** Notch signaling crosstalks with Wnt pathway, inhibited by γ-secretase inhibitors.

Molecular insights have implicated the *Wnt*/β-catenin signaling pathway as a crucial player in the pathogenesis of desmoid tumors, with almost all desmoids showing increased expression of β-catenin [1]. In the canonical *Wnt* signaling pathway, *APC* tightly regulates β-catenin levels by mediating its phosphorylation and subsequent degradation (Figure 1A). When Wnt binds to the cellular membrane, Axin translocates to the plasma membrane and disrupts the β-catenin destruction complex. (Figure 1B). In the presence of germline (FAP) or somatic mutations in *APC*, loss-of-function of the *APC* protein prevents proper degradation of β-catenin, leading to its accumulation in the cell and stimulation of cell proliferation through Tcf4 [2] (Figure 1C). For non-FAP associated sporadic cases, activating mutations of the β-catenin gene (*CTNNB1*) are widespread, with studies showing a 92% mutation rate in non-*APC* mutated desmoids [3]. β-catenin is also highly expressed in the proliferative phase of wound healing, and mouse models have demonstrated that overexpression of β-catenin is sufficient to cause development of desmoids [4]. In summary, the Wnt/β-catenin signaling cascade represents a common pathway through which multiple genetic alterations (*APC, CTNNB1*) and environmental dysregulations (wound-healing) converge to promote desmoid formation (Figure 1A-1C).

Multiple studies have demonstrated cross talk between the Notch signaling and the Wnt/β-catenin pathways [5, 6] (Figure 1D). Along with overexpression of β-catenin, desmoid tumors have been shown to highly express *NOTCH1* and its downstream transcription factor *HES1* [7]. γ-secretase is a key participant in Notch signaling through cleavage of the Notch intracellular domain (NICD), with subsequent translocation of NICD to the nucleus where it activates gene transcription. *In-vitro* studies of a γ-secretase inhibitor (PF-03084014) on desmoid tumor cell lines have shown dose-dependent decreases in expression of NICD and *HES1* with subsequent inhibition of cell line growth, migration, and invasion [7]. Differential gene expression analysis revealed upregulation of a critical Wnt/β-catenin gene, *WISP2*, suggesting *WISP2* as the link between both pathways in desmoid tumors.

Given the benign yet unpredictably locally progressive nature of desmoid tumors, initial treatment decisions are individualized and must take into account trajectory of tumor growth, surgical morbidity of tumor location, and patient symptomatology. A period of watchful waiting can be appropriate for patients who have stable, asymptomatic disease. When treatment is feasible and necessary, complete surgical excision with negative margins is the standard of care. Desmoids are also relatively radiosensitive and a course of radiation therapy (50-60Gy) is reasonable in patients who are not surgical candidates. Systemic therapy has shown responses in desmoids although the benign nature of the disease must be weighed against risks of toxicity. Generally, initial treatment is with less toxic agents such as nonsteroidal anti-inflammatory drugs (NSAIDs), tamoxifen, and imatinib, with cytotoxic chemotherapy reserved for more aggressive, organ compromising tumors. Recently, drugs have been developed to target the Notch pathway and have been evaluated in patients with desmoids. In a recent phase II trial of the γ-secretase inhibitor PF-03084014 in patients with pre-treated, progressive, symptomatic desmoid tumors, promising efficacy was observed with a partial response rate of 29% that lasted for more than 2 years [8]. In fact, all but one patient had a measurable regression of tumor volume. Of note, three of the five patients with partial responses experienced regression almost 2 years after the start of treatment. This correlates with the *in vitro* studies of PF-03084014 in desmoid cell lines which demonstrated that inhibition of the Notch pathway induced cell cycle arrest rather than directly causing cell apoptosis and death [7].

Desmoids represent a rare tumor that, although benign, can be locally aggressive and require treatment. Surgical excision remains the standard of care although newer approaches are needed for patients with unresectable, symptomatic desmoids. Further studies with novel agents that target the Notch and Wnt/β-catenin signaling pathways represent a promising potential treatment option for patients with desmoids.
